# The Impact of *Aspergillus fumigatus* Viability and Sensitization to Its Allergens on the Murine Allergic Asthma Phenotype

**DOI:** 10.1155/2013/619614

**Published:** 2013-08-26

**Authors:** Sumali Pandey, Scott A. Hoselton, Jane M. Schuh

**Affiliations:** Department of Veterinary and Microbiological Sciences, North Dakota State University, Fargo, ND 58108, USA

## Abstract

*Aspergillus fumigatus* is a ubiquitously present respiratory pathogen. The outcome of a pulmonary disease may vary significantly with fungal viability and host immune status. Our objective in this study was (1) to assess the ability of inhaled irradiation-killed or live *A. fumigatus* spores to induce allergic pulmonary disease and (2) to assess the extent to which inhaled dead or live *A. fumigatus* spores influence pulmonary symptoms in a previously established allergic state. Our newly developed fungal delivery apparatus allowed us to recapitulate human exposure through repeated inhalation of dry fungal spores in an animal model. We found that live *A. fumigatus* spore inhalation led to a significantly increased humoral response, pulmonary inflammation, and airway remodeling in naïve mice and is more likely to induce allergic asthma symptoms than the dead spores. In contrast, in allergic mice, inhalation of dead and live conidia recruited neutrophils and induced goblet cell metaplasia. This data suggests that asthma symptoms might be exacerbated by the inhalation of live or dead spores in individuals with established allergy to fungal antigens, although the extent of symptoms was less with dead spores. These results are likely to be important while considering fungal exposure assessment methods and for making informed therapeutic decisions for mold-associated diseases.

## 1. Introduction

For centuries, fungi have been associated with asthma and other airway diseases [1]. Humans inhale viable and nonviable fungi or their components in many indoor and outdoor environments, and mold-related exposures can pose a significant concern to human health [2–5]. Although a number of federal agencies provide guidance to the public on health effects associated with mold exposure and on ways to mitigate it, the United States Government Accountability Office (US GAO) reported a lack of federally accepted health-based standards for safe mold levels [6]. The problem is particularly concerning in postflooding or posthurricane situations and in agricultural settings where the repeated inhalation of mold over an extended period of time is likely [7–10]. 

*Aspergillus fumigatus* is one of the commonly detected fungal species in flooded indoor environments [11–14] and in grain dust [8, 15]. *A. fumigatus* has evolved to provide carbon and nitrogen turnover in decaying organic matter. However, due to their small size (2-3 *μ*m in diameter) and hydrophobicity, the spores (conidia) may remain suspended in the air for a long time, increasing the likelihood of inhalation deep into the alveolar spaces of human lungs. Exposure to *A. fumigatus* spores is ubiquitous and symptomless for most people, but it causes a spectrum of diseases in susceptible hosts. While invasive aspergillosis is a serious disease that may occur in immunocompromised individuals, most fungal diseases are associated with less mortality but an ongoing morbidity as is the case with allergic diseases in humans [16]. 

Several pulmonary diseases have been associated with *A. fumigatus*, such as allergic bronchopulmonary aspergillosis, severe asthma with fungal sensitization, rhinitis, sinusitis, and hypersensitivity pneumonitis. Although *A. fumigatus* is a source of 23 listed allergens [17] and is an opportunistic pathogen, *A. fumigatus*-induced pulmonary diseases may or may not involve elevated serum IgE or fungal colonization [18–20]. A meta-analysis study showed 15–48% prevalence rate for *Aspergillus *sensitization in bronchial asthma [21]. Besides being allergenic, research has provided evidence for secreted proteases [22–24] and cell wall-associated molecules, such as *β*-glucan [25–27] and chitin [28, 29], in orchestrating the host response to inhaled *Aspergillus*.

 As our understanding of the host pathogen interaction in the genesis of an *A. fumigatus*-induced pulmonary disease is emerging, models that mimic natural human exposure are critical. In the environment, humans inhale dry, airborne *A. fumigatus*. To mimic the exposure in experimental animals, invasive and noninvasive methods have been employed, such as intratracheal (IT), intranasal (IN), and inhalational (INH). Depending on the method used, the deposition, clearance [30], and stimulation of host immune responses to the substance intended for pulmonary delivery varies substantially [31, 32]. Besides being invasive, IT delivery results in a concentrated central deposition in the upper respiratory tract, where mucociliary clearance is predominant. Additionally, the IN and IT methods require suspension of the fungal spores in a liquid, which can considerably alter the spore coat, the concentration of soluble fungal antigens, and metabolic activity of the fungus [33]. Detergents such as Tween-80, which is used for the suspension of fungal spores for IT delivery, may influence host pathogen interaction by damaging the host epithelial cells [34] and/or by influencing fungal properties [35]. In contrast, the noninvasive INH method allows for repeated exposure to the same substance (as would occur in humans) and results in a dissemination of conidia throughout the lungs. In the INH method, no suspension of the conidia is required. 

 Inhalation of environmental substances is ubiquitous and an unavoidable phenomenon. While a healthy lung remains remarkably tolerant to inhaled antigens [22, 36, 37], allergy is a genetic predisposition to develop lung and systemic hypersensitivity reactions to environmental antigens (environmental allergies). This suggests that responses to inhaled substances can substantially vary in healthy and diseased states. Indeed, allergy is one of the strongest risk factors for acquiring asthma [38–40]. 

 Our objective in this study was twofold: (1) to assess the ability of inhaled irradiation-killed or live *A. fumigatus* spores to induce allergic pulmonary disease and (2) to assess the extent to which inhaled dead or live *A. fumigatus* spores influence pulmonary symptoms in a previously established murine allergic state. For this purpose, we used an inhalational apparatus that has been developed in our laboratory for the delivery of dry, aerosolized irradiation-killed (moisture-, heat-, and pressure-free sterilization method) or live airborne spores [41]. Previous studies comparing host immune responses to live and dead (typically killed by autoclaving) *A. fumigatus* conidia are not only limited by an unnatural route of human exposure but also exhibit variable inflammation and proallergic responses [26, 36, 42–44]. Moreover, reports comparing the pulmonary histopathological changes associated with live or dead *A. fumigatus* conidia are lacking. Studies, such as the one presented here, are likely to aid in establishing evidence-based standards for environmental mold exposures and remediation, as well as informing decisions for mold-associated pulmonary disease diagnoses, prognoses, and therapeutic interventions. 

## 2. Methods

### 2.1. Animals

BALB/c mice were obtained from the Jackson Laboratory (Bar Harbor, Maine, USA), and housed in a specific pathogen-free murine colony at Van Es Hall, North Dakota State University (NDSU, Fargo, ND, USA) in microfilter-topped cages (Ancare, Bellmore, NY, USA). Murine groups challenged with irradiated or live *A. fumigatus* conidia were caged separately. The study was conducted under the guidelines and approval of the Institutional Animal Care and Use Committee of NDSU.

### 2.2. Inhalation of Irradiation-Killed or Live *A. fumigatus* in a Nonsensitized or Allergically Sensitized Murine Host

**Figure 1:**
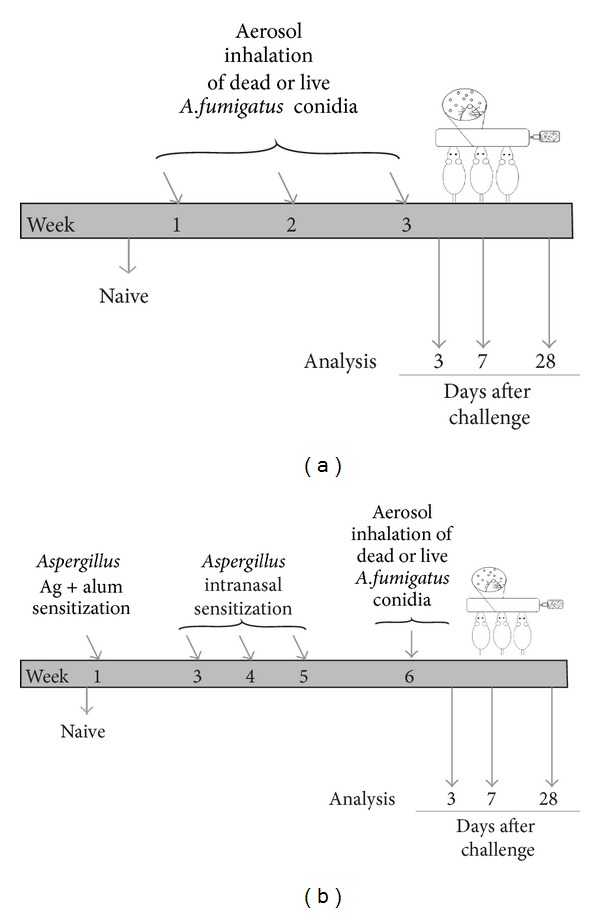
Schematic representation of the inhalation (INH) challenge protocol with irradiation-killed or live *Aspergillus fumigatus *conidia in a nonsensitized (a) or sensitized (b) murine host. For protocol (a), mice without prior fungal exposure were anesthetized with an intraperitoneal (IP) injection of ketamine and xylazine and subjected to a 10 min, nose-only INH of dry, aerosolized, dead, or live *A. fumigatus *conidia, once a week for three consecutive weeks. For protocol (b), mice were sensitized to fungal antigens by subcutaneous and IP injection of *A. fumigatus* antigen mixed with alum in PBS. This was followed by three weekly intranasal inoculations of *A. fumigatus* antigen in PBS. A week later, mice were challenged once a week for three consecutive weeks, with dead or live *A. fumigatus *conidia, in exactly the same way as for Protocol (a). Challenged mice treated according to protocol (a) or (b) were analyzed on days 3, 7, and 28 after third challenge. Naïve animals were maintained as negative controls.

For protocol (a) (Figure 1(a)), mice without prior exposure to fungal antigens (nonsensitized murine host) were challenged with airborne, dry, irradiation-killed, or live *A. fumigatus *conidia, using a previously described inoculation chamber and spore delivery method [41]. For *A. fumigatus *(strain NIH 5233; American Type Culture Collection) cultures, fresh fungal culture was spread onto sterile Sabouraud dextrose agar in a 25 cm^2^ culture flask and incubated at 37°C for 8 days. A separate aliquot was used for each fungal culture flask to ensure an equal yield of mature conidia. For dead conidia, the 8-day-old *A. fumigatus *culture flask was subjected to a lethal dose of gamma radiation (8 kGy) in a ^137^Cs gamma irradiator (Radiation Machinery Corporation, Parsippany, NJ, USA). Mice were anesthetized with an intraperitoneal (IP) injection of ketamine (75 mg/kg) and xylazine (25 mg/kg) prior to administration of a 10 min, nose-only inhalation (INH) of dead or live *A. fumigatus *conidia. To mimic repeated INH of an environmental allergen in humans, mice were challenged once a week for three weeks. 

For protocol (b) (Figure 1(b)), mice were sensitized to fungal extracts prior to challenge with dead or live *A. fumigatus* conidia. Mice were sensitized by subcutaneous and IP injection of 10 *μ*g of soluble *A. fumigatus *antigen (Greer Laboratories, Lenoir, NC) suspended in 0.1 mL Imject Alum (Pierce, Rockford, IL, USA) and 0.1 mL PBS. Two weeks after the injections, each mouse received a series of three, weekly 20 *μ*g intranasal (IN) inoculations consisting of soluble *A. fumigatus *antigen (Greer Laboratories, Lenoir, NC, USA) dissolved in 20 *μ*L PBS. One week after the last IN inoculation, mice were challenged with a 10 min, nose-only INH of dead or live *A. fumigatus *conidia, as in protocol (a). Mice were challenged once a week for three weeks and samples were collected at days 3, 7, and 28 after third fungal challenge. Naïve animals that were neither sensitized to fungal extract nor challenged with dead or live *A. fumigatus* conidia were maintained as baseline controls for the study. All fungal work was conducted in Class II biological safety cabinet, with the prior approval of the Institutional Biosafety Committee of NDSU.

### 2.3. Differential Cell Counts

The trachea was cannulated, and 1 mL of sterile PBS was used to lavage the bronchoalveolar space of the mouse. Total and differential cell counting was performed as previously described [41]. Representative photomicrographs were obtained using a Zeiss Z1 AxioObserver inverted microscope (Carl Zeiss Microscopy LLC, Thornwood, NY, USA).

### 2.4. Serum and BALF Antibody Analysis

Serum and BALF samples were obtained as previously described [41]. Mouse isotype-specific ELISA kits were used for quantification of IgA, IgG_1_, IgG_2a_ (Bethyl Laboratories Inc., Montgomery, TX, USA), and IgE (BD Biosciences, Inc., San Jose, CA, USA) antibody levels as per manufacturers' directions. For protocol (a), the serum was diluted as follows: IgA 1 : 1000, IgG_1_ 1 : 2500, IgG_2a_ 1 : 2500, and IgE 1 : 20, and the BALF was diluted as follows: IgA 1 : 5, IgG_1_ 1 : 10, IgG_2a_ 1 : 10, and undiluted for IgE analysis. For protocol (b), the serum was diluted as follows: IgA 1 : 1000, IgG_1_ 1 : 5000, IgG_2a_ 1 : 5000, and IgE 1 : 100, and the BALF was diluted as follows: IgA 1 : 10, IgG_1_ 1 : 20, IgG_2a_ 1 : 20, and IgE 1 : 2. 

### 2.5. cDNA Synthesis

The inferior and postcaval lobes of the lung were collected at each time point, snap frozen in liquid nitrogen, and stored at −20°C until use. Total RNA extracted from the homogenized lung tissues using TRIzol reagent (Invitrogen Life technologies, Grand Island, NY, USA) was subjected to DNase (Promega, Madison, WI, USA) (1 unit DNase/*μ*g RNA) treatment for 30 min at 37°C. RNA yield was determined by measuring the absorbance of ultraviolet light at 260 nm using a Synergy HT plate reader (BioTek Instruments Inc., Winooski, Vermont, USA). In order to prime the synthesis of first strand of cDNA by reverse transcription, 0.5 *μ*g of random primers (Promega, Madison, WI, USA) per *μ*g of RNA were allowed to anneal to the DNase-treated RNA at 70°C for 5 mins. Up to 2 *μ*g of RNA was used for each reaction. The RNA was reverse transcribed using Moloney Murine Leukemia Virus Reverse Transcriptase (M-MLV RT) and dNTP mix (Promega, Madison, WI, USA) at 37°C for 1 h. The reaction was stopped by heat inactivation at 70°C for 10 mins. The cDNA was used for analyzing the gene expression via quantitative PCR (qPCR), as mentioned later. 

### 2.6. qPCR

The expression of *ccl-17, tslp*,  and *hprt-1 *(internal control) genes in murine lungs was analyzed by qPCR using SYBR green-based master mix and RNA-specific QuantiTect primer assays (QIAGEN, Valencia, CA, USA) for mouse. The reaction was set up on ABI 7500 real-time PCR machine (Applied Biosystems, Carlsbad, CA, USA): 95°C for 10 min (activation of HotStar Taq DNA Polymerase), 95°C for 15 secs (denaturation), and 60°C for 1 min (annealing and extension). The denaturation, annealing, and extension cycles were repeated 40 times, and the fluorescence data was collected at the end of each cycle. Dissociation curves analysis was performed, and the data was analyzed using 2^−ΔΔCT^ method to calculate the relative fold change in the lung, standardized against naïve controls. A four-point dilution curve was established to show the validity of 2^−ΔΔCT^ calculations prior to use for analysis and to determine the appropriate dilution of cDNA to be used for real-time qPCR reaction. 

### 2.7. Histological Analysis

Whole left lungs were fixed in 10% neutral buffered formalin and paraffin embedded. 5 *μ*m thick lung sections were affixed to microscope slides and stained with hematoxylin and eosin (H&E) stain (Dako North America Inc., Carpinteria, CA, USA), periodic acid Schiff (PAS) stain, or Gomori's trichome stain (both from Richard Allan Scientific Inc., Kalamazoo, MI, USA) to assess pulmonary inflammation, goblet cell (GC) metaplasia, and collagen deposition in the lung, respectively. Representative photomicrographs were obtained using a Zeiss Z1 AxioObserver inverted microscope (Carl Zeiss Microscopy LLC, Thornwood, NY, USA). 

A photometric analysis using Olympus MicroSuite software (Olympus America Inc., Center Valley, PA, USA) was employed to analyze the histology images. The PAS-positive mucus-producing GCs were counted in 5 randomly selected 200 *μ*m segments of basement membrane in the lateral bronchial branches or small airways of mice lungs. The percentage of GC to total columnar epithelial cells was calculated for each group. The H&E-stained sections were used to measure the thickness of columnar epithelial cells lining the airways. At least 50 discrete points at 50 *μ*m intervals were selected along the second or third lateral (L2 or L3) bronchial branch and a perpendicular line extending from the basement membrane was drawn through the cell to the height of epithelial cell layer thickness. For subepithelial collagen deposition, at least 100 discrete points at 50 *μ*m intervals were selected along bronchial branch L2 or L3, and a perpendicular line was drawn from a point on the basement membrane through the full thickness of the peribronchial collagen. Group mean was determined by averaging the mean for each mouse in a group. 

### 2.8. Immunohistochemistry

Longitudinal lung sections of left lungs affixed to glass slides were deparaffinized and placed in citric acid buffer (10 mM, pH 6.0) and cooked in a microwave pressure cooker (Nordic Ware, Minneapolis, MN, USA) for 10 min for antigen retrieval. IgA, IgE, and IgG (primary antibodies from Southern Biotech, Birmingham, AL, USA) staining was performed using horseradish peroxidase (HRP)-3-amino-9-ethylcarbazole (AEC) cell and tissue staining kit (R&D Systems, Minneapolis, MN, USA), as per manufacturer's recommended protocol. Red colored precipitates were identified as areas of positive staining in the lung. Representative photomicrographs were obtained using a Zeiss Z1 AxioObserver inverted microscope (Carl Zeiss Microscopy LLC, Thornwood, NY, USA).

### 2.9. Statistical Analysis

All results are expressed as the mean ± the standard error of mean. GraphPad Prism 5 software (GraphPad Software, Inc., LaJolla, CA, USA) was used to calculate statistics; differences between groups were tested with two-tailed unpaired Student's *t*-test with Welch's correction. In all cases, *P* < 0.05 was considered statistically significant. 

## 3. Results

### 3.1. Effect of Live and Irradiation-Killed *A. fumigatus* Inhalation on Allergy-Associated Responses

**Figure 2:**
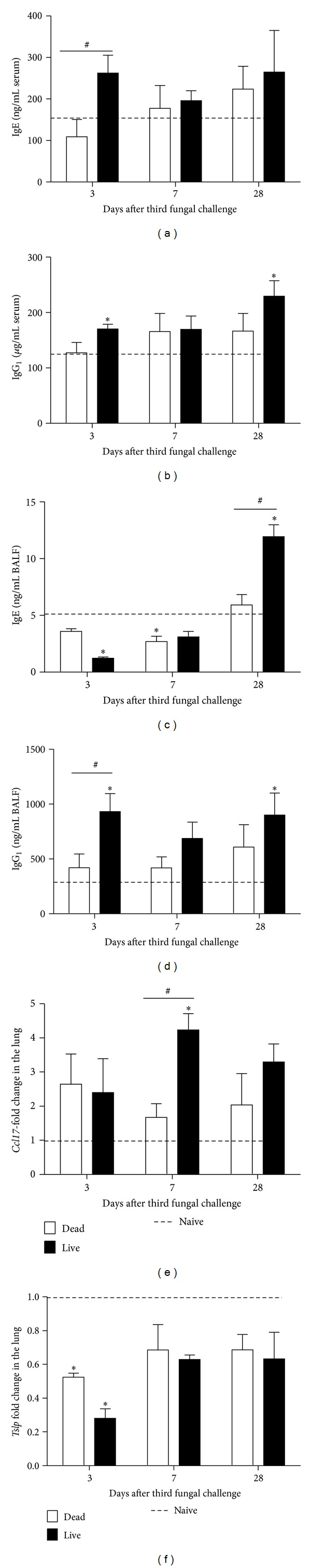
Effect of inhaled dead or live *A. fumigatus* conidia on the development of allergy-associated responses in a nonsensitized murine host. Total IgE (a, c) and IgG_1_ (b, d) levels were determined in the serum (a, b) and bronchoalveolar lavage fluid (BALF) (c, d) samples obtained from mice challenged with dead or live *A. fumigatus *conidia, at days 3, 7, and 28 after third fungal challenge. Commercially available mouse-specific ELISAs were used for this purpose. *Ccl17* (e) *and tslp* (f) mRNA levels were determined in the murine lung homogenates, via SYBR green-based quantitative polymerase chain reaction. The fold change in the lung was determined using 2^-ΔΔCT^ method and standardized against naïve levels (dashed line). Bars represent mean ± SEM, *n* = 3–5 mice/group. *, ^#^, *P* value < 0.05, as compared with naïve mice or mice challenged with dead conidia, respectively.

Our first objective was to assess the extent to which the inhalation of dead or live *A. fumigatus *conidia could induce allergy in a non-sensitized murine host (Protocol (a), Figure 1(a)). In experimental animal models, allergy is typically assessed by elevated antibody levels (IgE and IgG_1_). We found that repeated inhalation of dead or live *A. fumigatus* conidia did not result in elevated serum IgE levels, compared to naïve mice (Figure 2(a)). Similar results were observed in three repeats of the study. However, the serum IgG_1_ levels were elevated in mice challenged with live but not dead conidia, at days 3 and 28 after third fungal challenge, compared to naïve mice (Figure 2(b)). The BALF antibody levels were measured to assess the local effect of inhaled conidia on the humoral immune response. Although a decrease was observed at earlier timepoints, by day 28, the BALF IgE levels in mice challenged with live conidia showed a ~2-fold increase over naïve levels (Figure 2(c)). Similarly, an elevation in BALF IgG_1_ was observed at days 3 and 28, in mice challenged with live but not the dead conidia (Figure 2(d)). 

Since allergy is believed to be a Th-2-dominant process, we analyzed the mRNA profiles of two Th-2 associated markers: *ccl17* and *tslp*. At day 7, mice challenged with live but not the dead conidia showed elevated *ccl17 *mRNA levels (Figure 2(e)). At day 3, the *tslp* mRNA levels showed an unexpected decrease in mice challenged with dead or live conidia, compared to naïve mice (Figure 2(f)). Although the reason for the later finding is not immediately apparent, the elevation in BALF IgE, IgG_1_, and lung *ccl17* mRNA levels with live conidia inhalation suggests that viable conidia have a greater tendency to induce allergy than the nonviable conidia (Figure 2). 

### 3.2. Inhalation of Live *A. fumigatus* Conidia Elicits Mucosal Antibody Response

**Figure 3:**
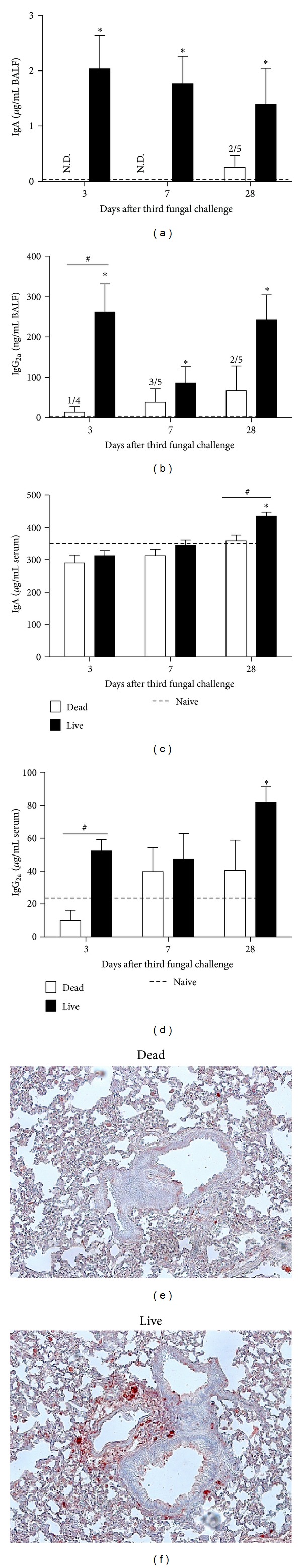
Effect of inhaled dead or live *A. fumigatus* conidia on IgA and IgG_2a_ levels in the serum, BALF, and lung sections of a non-sensitized murine host. Total IgA (a, c) and IgG_2a_ (b, d) levels were determined in the BALF (a, b) and serum (c, d) samples obtained from mice challenged with dead or live *A. fumigatus *conidia, at days 3, 7, and 28 after third fungal challenge. Commercially available mouse-specific ELISAs were used for this purpose. Immunohistochemical staining for IgA antibody localization on lung sections obtained from mice challenged with dead (e) or live (f) *A. fumigatus* conidia, at day 7 after third fungal challenge, showed cell-associated IgA in mice challenged with live *A. fumigatus* conidia (e). IgE and IgG (not shown) positive immunohistochemical staining showed secreted- or endothelium-associated antibody in these mice. Bars represent mean ± SEM, *n* = 4-5 mice/group. * and ^#^ designate a *P* < 0.05, as compared with naïve mice or mice challenged with dead conidia, respectively. Scale bar = 100 *μ*m.

Previous study with live and heat-killed *A. fumigatus* conidia assessed the antibody levels in the serum but not in the BALF [43]. Since dissociated trends for serum and BALF IgE and IgG_1_ antibody levels were observed with inhalation of live *A. fumigatus* conidia, we investigated the systemic and local levels of other antibody isotypes in these mice. The naïve mice had undetectable levels of BALF IgA (Figure 3(a)) and IgG_2a_ (Figure 3(b)). With the exception of sporadic, low-level detection in a few mice (fraction on the graph = no. of positives/total no. of mice in a group), the mice challenged with dead conidia had BALF IgA and IgG_2a_ levels similar to naïve levels (Figures 3(a) and 3(b)). In contrast, the mice challenged with live conidia had significantly increased BALF IgA and IgG_2a_ levels at days 3, 7, and 28 (Figures 3(a) and 3(b)). The mice challenged with dead conidia had detectable levels of serum IgA and IgG_2a_, and the levels were similar to naïve mice (Figures 3(c) and 3(d)). In contrast, by day 28, the serum antibody levels of IgA (Figure 3(c)) and IgG_2a_ (Figure 3(d)) were significantly elevated in mice challenged with live conidia. The effect of live conidia inhalation on the BALF antibody levels was prominent as early as day 3 after fungal challenge. However, a significant elevation in serum antibody levels was not observed until day 28 after fungal challenge, suggesting a predominantly local effect and a delayed systemic effect of inhaled live *A. fumigatus* conidia on the humoral immune response in these mice (Figures 3(a)– 3(d)). 

To further assess the spatiotemporal distribution of antibodies, immunohistochemical (IHC) staining was performed on murine lungs challenged with dead or live *A. fumigatus* conidia. The murine lung sections incubated with buffer without primary antibody were maintained as negative controls and consistently stained negative for red precipitates (not shown). The IHC identified an abundance of cell-associated IgA in the parenchyma and the peribronchovascular region of the murine lungs challenged with live conidia (Figure 3(f)), but not the dead conidia (Figure 3(e)) at days 3, 7, and 28 after third fungal challenge. A peak in IgA positive staining was observed at day 7 and is depicted in Figures 3(e) and 3(f). In contrast to IgA, IgE, and IgG positive staining was not cell associated but mainly appeared as secreted, lining the endothelium of the blood vessels around the large airways (data not shown). While increased IgE positive staining was observed in lung sections obtained from mice challenged with live conidia, IgG positive staining was similar in both groups. Based on our observations, it would be reasonable to expect the presence of IgA-producing cells in the lungs and an extrapulmonary source for IgE and IgG. 

### 3.3. Live Conidia Inhalation Elicits Significantly Increased Granulocytic Pulmonary Inflammation

The lung sections stained with H&E or cytospinned BALF samples stained with a differential stain were analyzed to assess total and differential inflammation in the mice challenged with dead or live *A. fumigatus* conidia.

**Figure 4:**
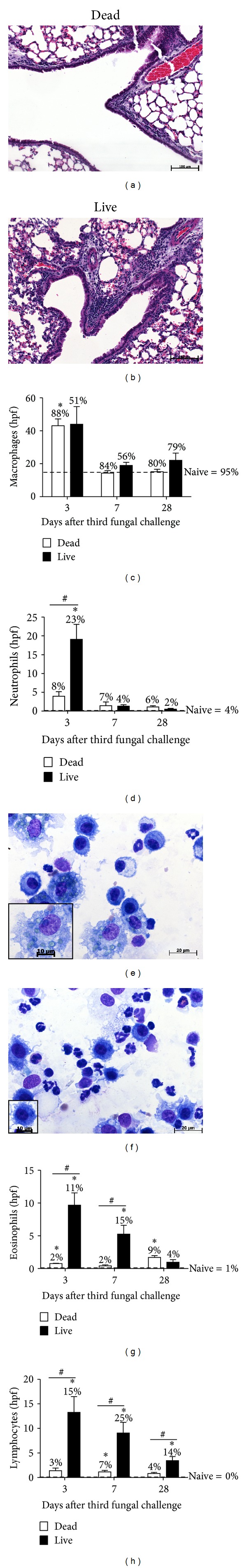
Effect of inhaled dead or live *A. fumigatus* conidia on pulmonary inflammation in non-sensitized mice. Peribronchovascular inflammation observed on hematoxylin and eosin (H&E) stained lung sections peaked at day 3, for mice challenged with dead (a) or live (b) *A. fumigatus* conidia, is depicted here. BALF samples from naïve mice or mice challenged with dead or live *A. fumigatus* conidia were cytospun and stained with Quick Dip stain to aid in the analysis of differential cell types based on their morphology and staining pattern. Monocytes/macrophages (c), neutrophils (d), eosinophils (g), and lymphocytes (h) were enumerated for each group at days 3, 7, and 28 after third fungal challenge. Counts from naïve mice are represented by a dashed line (c, d, g, and h). The percentages indicated on the bars in (c), (d), (g), and (h) represent the cells quantified as % of total cells. At day 3, significant differences in the number of granulocytes were observed in the mice challenged with dead (e) or live (f) conidia. Additionally, macrophages (shown in the inset) from the two groups appeared morphologically different. The differences in the size of the insets indicate differences in size of the macrophages shown in the insets. Macrophages from dead conidia-challenged mice had numerous greenish spherical objects, presumably conidia, inside them (e inset), and these were rarely observed in the macrophages from live conidia-challenged group (f inset). Bars represent mean ± SEM, *n* = 4-5 mice/group. *, ^#^, *P* value < 0.05, as compared with naïve mice or mice challenged with dead conidia, respectively. Scale bars = 100 *μ*m (a, b), 20 *μ*m (e, f), and 10 *μ*m (e, f insets).

The naïve mice did not show any peribronchovascular inflammation in the H&E stained lung sections (not shown). At all the tested time points, the incidence and severity of pulmonary histopathology was significantly less in mice challenged with dead conidia (Figure 4(a)) than in mice challenged with live conidia (Figure 4(b)). Representative pictures at day 3 after challenge with dead or live conidia are depicted in Figures 4(a) and 4(b), respectively. In the mice challenged with live conidia, the inflammation was most intense at day 3 after challenge (Figure 4(b)), and although a decrease in inflammation was seen at day 7, it persisted even at day 28 after challenge. In the mice challenged with dead conidia, the inflammation was resolved by day 7. The peribronchovascular inflammation was centered around large diameter, main axial conducting airway and extended into the more distal, small diameter, and terminal bronchioles. At all the time points, the inflammatory influx was comprised predominantly of mononuclear inflammatory cell infiltrate composed mainly of large and small lymphocytes and monocytes. 

In the BALF obtained from naïve mice (dashed line in Figures 4(c), 4(d), 4(g), and 4(h)), macrophages were the only cell type detected, and even after challenge with dead or live conidia, macrophages remained the predominant cell type (88% for dead group and 51% for live group) at all time points (Figure 4(c)). Although both dead (*P* value < 0.05) and live conidia (*P* value = 0.06) recruited macrophages at day 3 after challenge (Figure 4(c)), distinct differences were noted in the appearance of the macrophages obtained in the cytospinned BAL samples from the two groups (Figures 4(e) and 4(f) insets). The cytoplasm of the macrophages obtained in the BAL of mice challenged with dead conidia looked enlarged, foamier, and hypervacuolated as compared with those obtained in the BAL from mice challenged with live conidia. Greenish spherical objects, which are presumably the intact conidia, could be observed more frequently in the macrophages of the dead group. On average, 10 greenish spherical objects per macrophage (range = 0 to 28) were counted from the dead group compared with an average of 1 greenish spherical object per macrophage (range = 0 to 7) observed in the live group. Although the true identity of these greenish spherical objects remain obscure right now, their consistent presence in the macrophages obtained from the BAL of mice challenged with dead *A. fumigatus* conidia is certainly intriguing and a focus of future investigation.

Mice challenged with live conidia recruited a significantly greater number of neutrophils (Figure 4(d)), eosinophils (Figure 4(g)), and lymphocytes (Figure 4(h)), compared to mice challenged with dead conidia. Although mice challenged with dead conidia showed sporadic presence of eosinophils, the number (~1-2) was too low to significantly contribute towards the inflammatory profile at the tested time points (Figure 4(g)). 

Taken together, this data suggests that the inhalation of live *A. fumigatus* conidia results in significantly greater pulmonary inflammation, compared to the inhalation of dead conidia or in the absence of fungal challenge (Figure 4). 

### 3.4. Enhanced Airway Remodeling Is Observed with Live *A. fumigatus* Conidia Inhalation

**Figure 5:**
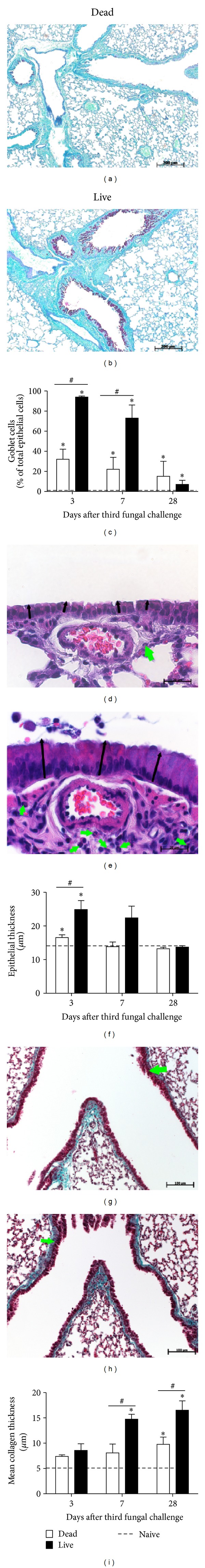
Effect of inhaled dead or live *A. fumigatus* conidia on airway remodeling in non-sensitized murine host. Periodic acid-Schiff (PAS) staining (a, b), H&E staining (d, e), and Gomori's trichrome staining (g, h) were used to measure goblet cell% lining the columnar epithelial cells (c), epithelial layer thickness (f), and subepithelial collagen layer thickness (i), respectively. Green arrows are pointing towards the granulocytes in the peribronchovascular region (d, e) or subepithelial collagen deposition and smooth muscle thickness (g, h). Naïve levels are indicated by the dashed line (c, f, i). Bars represent mean ± SEM, *n* = 3–5 mice/group. *, ^#^, *P* value < 0.05, as compared with naïve mice or mice challenged with dead conidia, respectively. Scale bars = 200 *μ*m (a, b), 20 *μ*m (d, e), and 100 *μ*m (g, h).

Airway remodeling is defined as structural change of the airway wall, which arises from injury and repair, and plays an important role in asthma pathophysiology by decreasing the lung function. The naïve levels for remodeling parameters such as GC metaplasia (Figures 5(a)– 5(c)), epithelial layer thickening (Figures 5(d)–5(f)), and subepithelial collagen deposition (Figures 5(g)–5(i)) are shown as a dashed line. Challenge with dead and live conidia led to an increase in GC% over naïve mice at days 3, 7, and 28 after third fungal challenge (Figure 5(c)). However, the increase was significantly greater with live conidia challenge (~3-fold increase at days 3 and 7) than with dead conidia challenge (Figure 5(c)). Additionally, live conidia led to significantly increased epithelial thickness at day 3 after challenge, as compared to dead conidia challenge or no challenge (Figure 5(f)). Furthermore, an increased number of granulocytes were identified based on morphology or red cytoplasmic staining in the subepithelial zone of mice challenged with live conidia at day 3, as compared to mice challenged with dead conidia (green arrows in Figures 5(d) and 5(e)). Correlating with increased granulocyte recruitment in the peribronchovascular region of murine lungs challenged with live conidia, we observed increased subepithelial collagen deposition (green arrows in Figures 5(g) and 5(h)) in these mice at days 7 and 28 (Figure 5(i)). 

### 3.5. Th-2-Associated Systemic and Mucosal Humoral Immune Responses Are Enhanced following Inhalation of Live *A. fumigatus* Conidia in Allergically Sensitized Mice

The next question we asked was: does an allergically sensitized murine host respond differently to dead and live *A. fumigatus* conidia inhalation? For this purpose, we sensitized the murine host to fungal extract antigens (Protocol (b), Figure 1(b)) prior to the three weekly challenges with dead or live *A. fumigatus* conidia. As described in previous studies from our lab, sensitization without challenge with *A. fumigatus* conidia (day 0 time point) leads to an increased humoral response, but the pulmonary inflammation and GC metaplasia remain similar to naïve mice [41, 45]. 

**Figure 6:**
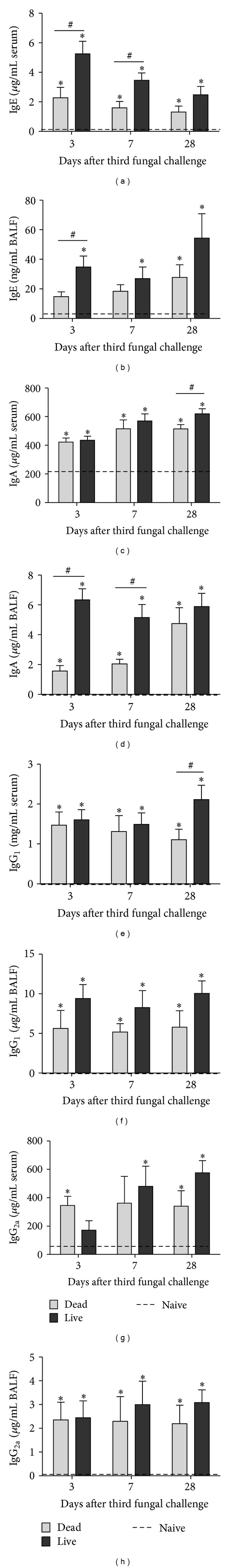
Effect of inhaled dead or live *A. fumigatus* conidia on systemic and mucosal antibody levels in a sensitized murine host. Total IgE (a, b), IgA (c, d), IgG_1_ (e, f), and IgG_2a_ (g, h) levels were determined in the serum (a, c, e, and g) and BALF (b, d, f, and h) samples obtained from mice challenged with dead or live *A. fumigatus *conidia, at days 3, 7, and 28 after third fungal challenge. Commercially available mouse-specific ELISAs were used for this purpose. Bars represent mean ± SEM, *n* = 4–8 mice/group. *, ^#^, *P* value < 0.05, as compared with naïve mice or mice challenged with dead conidia, respectively.

Due to the prior sensitization of mice to fungal extracts, the systemic and mucosal humoral immune response was significantly elevated in the sensitized mice, compared to naïve mice (Figure 6). To investigate if inhalation of dead or live *A. fumigatus* conidia can influence the established humoral immune response, we analyzed the immunoglobulin levels in the serum (Figures 6(a), 6(c), 6(e), and 6(g)) and BALF (Figures 6(b), 6(d), 6(f), and 6(h)). When compared to the inhalation of dead *A. fumigatus* conidia, exposure to live *A. fumigatus* led to a significant increase in serum IgE levels at days 3 and 7 (Figure 6(a)), BALF IgE levels at day 3 (Figure 6(b)), serum IgA (Figure 6(c)) and serum IgG_1_ (Figure 6(e)) at day 28, and BALF IgA at days 3 and 7 (Figure 6(d)). In contrast, the BALF IgG_1_ (Figure 6(f)) and serum and BALF IgG_2a_ (Figures 6(g), 6(h)) antibody levels remained similar in mice challenged with live or dead conidia at all the tested time points. Taken together, our data suggests that compared to Th-1-associated humoral immune response (IgG_2a_), Th-2-associated humoral immunity (IgE and IgG_1_) and mucosal antibody (IgA) are more susceptible to an increase upon inhalation of live but not the dead, *A. fumigatus* conidia in a sensitized murine host (Figure 6). 

### 3.6. Significant Neutrophil Recruitment and Goblet Cell Metaplasia Is Observed in Allergically Sensitized Mice Challenged with Dead and Live *A. fumigatus* Conidia

**Figure 7:**
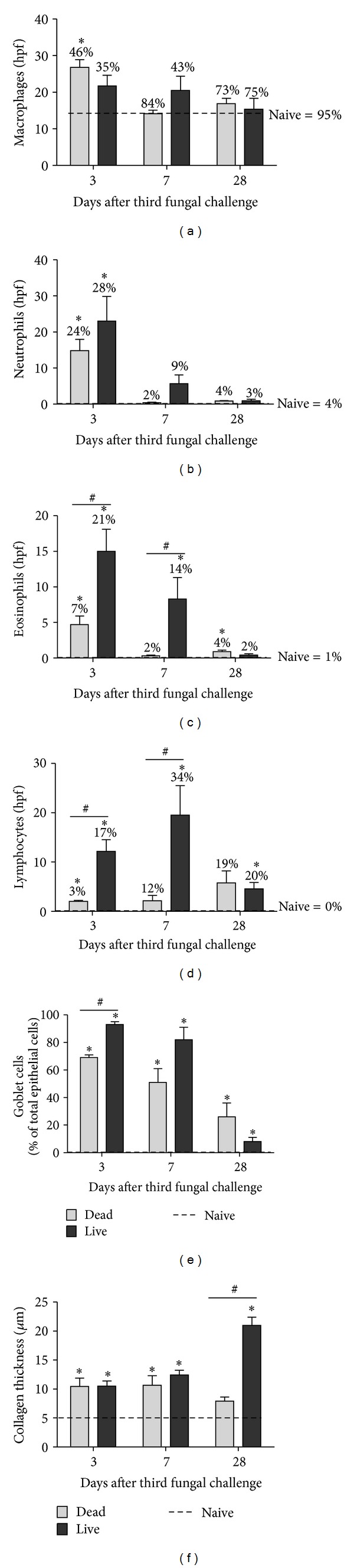
Effect of inhaled dead or live *A. fumigatus* conidia on pulmonary inflammation and airway remodeling in sensitized mice. BALF cells from naïve mice or mice challenged with dead or live *A. fumigatus* conidia were cytospun and stained with Quick Dip stain for differential analysis. Monocytes/macrophages (a), neutrophils (b), eosinophils (c), and lymphocytes (d) were enumerated for each group at days 3, 7, and 28 after third fungal challenge. The percentages indicated on the bars in (a–d) represent the cells quantified as % of total cells. PAS staining or Gomori's trichome staining was used to measure goblet cell% lining the columnar epithelial cells (e) or subepithelial collagen layer thickness (f). Naïve levels are indicated by the dashed line. Counts from naïve mice are represented by a dashed line (a–f). Bars represent mean ± SEM, *n* = 4–8 mice/group. * and ^#^ represent *P* < 0.05, as compared to naïve mice or mice challenged with dead conidia, respectively.

The analysis of differential cell types in the BALF showed that mice challenged with dead *A. fumigatus *conidia had significantly more macrophages compared to naïve mice at day 3 after third fungal challenge (Figure 7(a)). The differences in macrophage counts in mice challenged with live *A. fumigatus* conidia were not statistically significant, with respect to naïve mice (Figure 7(a)). Unlike the morphological differences observed in non-sensitized mice (Figures 4(e) and 4(f)), macrophages appeared the same in allergically sensitized mice challenged with either dead or live conidia. 

Compared to naïve mice, the inhalation of dead or live conidia recruited neutrophils by day 3, and there were no differences in neutrophil counts between mice challenged with live or dead *A. fumigatus* conidia (Figure 7(b)). At day 3 after challenge, mice challenged with dead conidia had elevated numbers of eosinophils (Figure 7(c)) and lymphocytes (Figure 7(d)). By day 7, the numbers were reduced to naïve levels. Mice challenged with live conidia had significantly more eosinophils (Figure 7(c)) and lymphocytes (Figure 7(d)) at days 3 and 7, as compared to mice challenged with dead conidia and naïve mice. 

The GC% was significantly elevated in mice challenged with dead and live *A. fumigatus* conidia, compared to naïve mice, at days 3, 7, and 28 after third fungal challenge (Figure 7(e)). In contrast to non-sensitized mice (Figure 5(c)), the inhalation of live conidia led to a modest (<1.5 fold) increase in GC%, compared to the inhalation of dead conidia, at day 3, but by day 7, both groups had similar levels (Figure 7(e)). The collagen thickness was increased in mice challenged with dead and live conidia at days 3 and 7, compared to naïve mice (Figure 7(f)). However, by day 28, a significant (~4 fold) increase in collagen thickness was observed in mice challenged with live conidia, compared to naïve mice and dead conidia-challenged mice (Figure 7(f)). Taken together, this data suggests that compared to the inhalation of dead conidia in a sensitized murine host, inhalation of live *A. fumigatus* conidia results in only a modest difference in GC% at earlier time points, but a significant increase in collagen deposition at day 28 after challenge.

## 4. Discussion

Although previous studies have reported differences in innate and adaptive immune responses to live and dead *A. fumigatus* conidia [26, 36, 42, 43], this is the first comparative study to have utilized inhalation as a route for pulmonary delivery of dry, aerosolized *A. fumigatus* conidia. We showed that in a non-sensitized murine host (Protocol 1(a)), live but not irradiation-killed *A. fumigatus* conidia inhalation elicited significantly more pulmonary inflammation, mucosal antibody production, and airway remodeling, which suggests that inhalation of live but not dead, *A. fumigatus* conidia has a greater tendency to elicit asthma symptoms. 

We also report a comparison of host immune response with repeated inhalation of live and dead *A. fumigatus* conidia in a murine host with prior sensitization to fungal extracts (Protocol 1(b)). In these mice, inhalation of live and dead *A. fumigatus* conidia elicited asthma symptoms, albeit the extent of response to dead conidia was lesser than that for live conidia. As described in previous studies from our lab, sensitization without challenge with *A. fumigatus* conidia (Day 0 time point) leads to an increased humoral response, but the pulmonary inflammation and GC metaplasia remain similar to naïve mice [41, 45]. Indeed, as a result of sensitization with *A. fumigatus *extract and alum, an increased eosinophil recruitment and systemic and mucosal humoral immune responses were observed, suggesting a Th-2 predominant response in sensitized mice as compared with their non-sensitized counterparts. However, striking differences were observed with regard to neutrophil recruitment and GC% in sensitized (Protocol 1(b)) versus non-sensitized (Protocol 1(a)) mice challenged with dead *A. fumigatus* conidia. The sensitized mice challenged with dead conidia recruited neutrophils (24% ± 5% of total BAL cells) at day 3 after challenge, which was similar to the sensitized mice challenged with live conidia (28% ± 6%) and significantly higher than non-sensitized mice challenged with dead conidia (8% ± 2%). Additionally, sensitized mice challenged with dead conidia showed significantly increased GCs (69% ± 2% of the total columnar epithelial cells) in the airways, at day 3 after challenge, compared with 32% ± 10% seen in non-sensitized mice challenged with dead conidia.

In experimental settings, allergy is typically assessed by elevated antibody levels (IgE and IgG_1_) [38, 40]. Although an elevation in serum IgE, with 4 or 8 intranasal challenges of live *A. fumigatus* conidia, has been reported previously [46], a significant increase in serum IgE was not observed in our study after three repeated inhalations of dead or live *A. fumigatus* conidia in non-sensitized mice. In accordance with our study, elevations in serum IgG_1_ but not IgE, with live *A. fumigatus* conidia have been observed previously [43]. The differences are likely to be due to the dosing regimen and exposure frequency. It has been previously proposed, that in contrast to T-cell responses, antibody-based methods lack sensitivity to detect allergy, at least in mice [47]. The problem is likely to exist in clinical settings as well, since not all asthma patients with fungal colonization, are detected as IgE positive and vice versa [19, 48]. Since the diagnosis may dictate the use of antifungal and/or anti-IgE therapy, a need for better diagnostic methods for assessing fungus-associated allergy/asthma exists. 

Elevated antibody levels in the BALF may play a critical role in host defense against respiratory pathogens and inhaled allergens. The elevated BALF antibody levels, with live but not the dead *A. fumigatus* conidia, starting as early as day 3 after challenge, suggest that lung mucosa is the primary target for inhaled *A. fumigatus* conidia. Both extrapulmonary (bone marrow or respiratory lymph nodes) and pulmonary sources of IgE have been identified [49], although no IgE^+^ cells were detected by IHC in the present study. Although the source of antibodies was not immediately apparent, our results are in alignment with studies in human ABPA patients, where elevated IgA, IgG, and IgE levels have been detected in the BALF [50]. 

The IgE levels in the BALF of non-sensitized mice challenged with live *A. fumigatus* conidia showed a ~2-fold elevation over naïve levels at day 28 after challenge. Although quantitatively the levels were still in nanograms, a couple of things are striking about this observation. Our data suggests that inhalation of live but not the dead, *A. fumigatus* conidia might play an important role in the development of allergic disease, in the human population. BALF but not the serum IgE levels showed early elevation, suggesting that the lung mucosa is the predominant site for *A. fumigatus*-induced IgE production and/or accumulation, in individuals exposed to live *A. fumigatus*. This would warrant against skin prick test to ascertain fungal sensitization. A similar observation has been previously reported in allergic rhinitis patients, who had allergen-specific IgE in nasal secretions and negative skin prick test response [51, 52]. Diagnostic standards related to BALF IgE levels, in order to determine fungal sensitization, do not exist. Since an accurate diagnosis dictates the administration of anti-IgE therapy, further research is needed to validate BALF IgE levels in fungal sensitization. 

A genetic predisposition to develop allergy is mostly associated with Th-2 predominant response. *A. fumigatus* proteases have been shown to promote a Th-2-type response [22], and it is a source of 23 listed allergens [17]. In previous studies, significant variations in total and Th-2-associated inflammatory responses to live and dead *A. fumigatus *have been observed. Rivera et al. reported that intratracheal (IT) challenge with dead conidia (heat-inactivated conidia; HIC) primed IL-4 and IL-13 producing CD4^+^T cells (proallergic) in the draining lymph nodes. In contrast, live conidia recruited IFN-*γ* producing CD4^+^T (counter regulatory for Th-2) cells to the airways [43]. Porter et al. reported that prolonged intranasal (IN) administration of dead (paraformaldehyde fixed)* A. fumigatus* conidia promoted significant lung IL-4 and eosinophil (proallergic) responses, although reduced compared to responses with live conidia [42]. Similar results were obtained with live and dead (irradiated) *A. niger* species in another study by Porter et al. [47]. In contrast, Aimanianda et al. showed that IN delivery of dead (dormant, paraformaldehyde fixed) *A. fumigatus* conidia elicited no inflammation in mice [36]. Hohl et al. also reported that dead (HIC) conidia were less stimulatory with regard to inflammation [26]. Finally, Murdock et al. reported coevolution of Th-1, Th-2, and Th-17 responses during repeated IN exposure to *A. fumigatus* conidia [46]. While Th-1 responses are likely to inhibit Th-2 responses, and the role of Th-17 immune response in asthma is still unclear, further investigations that mimic natural human exposure, such as the one presented here, are warranted. 

CCL17 promotes the recruitment of Th-2 cells into the airways [53], and fungal proteases have been implicated in upregulating CCL17 [54]. Moreover, elevated serum CCL17 levels have been proposed as a biomarker of allergic bronchopulmonary aspergillosis [55, 56]. Based on these findings, we hypothesized that live but not the dead *A. fumigatus* conidia challenge, due to *in situ* protease production, would induce *ccl17 *in non-sensitized mice. Indeed, *ccl17* mRNA upregulation with live but not the dead *A. fumigatus* conidia inhalation provided evidence in support of our hypothesis. Thymic stromal lymphopoietin (TSLP) is believed to be a master regulator of Th-2-driven inflammation [57, 58] and the allergic asthma phenotype [59]. Additionally, an *in vitro* study demonstrated increased TSLP in epithelial cells, with fungal (*Alternaria*) extracts [60]. This led us to hypothesize that challenge with live *A. fumigatus* conidia would lead to increased *Tslp* gene expression, indicative of a Th-2 dominant response. However, we found an unexpected decrease in *Tslp* mRNA expression with live and dead *A. fumigatus *conidia, at day 3 after fungal challenge. While the reason for the reduction is not immediately apparent, an *A. fumigatus*-specific effect on murine lung might be involved and is likely to be independent of fungal viability status. Interestingly, PAR-2 (mRNA), which is believed to be important in fungal (*Alternaria*) extract-mediated TSLP induction [60], is inhibited in a TLR4-dependent manner by *A. fumigatus*-infection in immunocompromised mice [61]. To our knowledge, this is the first documentation of *tslp* mRNA levels in an *A. fumigatus* induced pulmonary disease model, and further investigation is warranted. 

While mucus production is largely considered as an innate immune response in healthy people, the Th-2 predominant immune response in asthma patients results in chronic mucus hypersecretion [62, 63]. In the present study, a non-sensitized murine host challenged with live *A. fumigatus* conidia showed significantly increased mucus-producing goblet cells, compared with dead conidia, which suggested that mucus production was not only an innate immune response to particulate matter but can be significantly affected with the viability status of the inhaled substance. This raises a question regarding which fungal-associated factors might contribute to GC metaplasia in *in vivo* settings? Indeed, proteases produced by *A. fumigatus* can induce a Th-2 dominant response [22, 64], and specifically serine protease activity in *A. fumigatus* was found to be essential to induce the expression of the mucin producing *Muc5ac* gene and mucin secretion in the bronchial epithelial cells [65]. While these studies have established a critical role for proteases in *A. fumigatus*-induced mucus secretion, they have either utilized purified protease extracts and/or are *in vitro*. Therefore, the present study demonstrates the importance of factors associated with viable, dry, aerosolized conidia (mimicking natural human exposure), and potentially an *in situ* production of proteases, directly or indirectly promoting GC metaplasia. We also showed that GC metaplasia was significantly enhanced with the inhalation of both dead and live conidia in sensitized mice, suggesting that a pre-existing Th-2 predominant immunity in sensitized mice may also contribute to GC metaplasia, irrespective of fungal viability status. 

Although pulmonary fibrosis is an infrequently observed feature of human asthma, studies have linked the intensity of fibrosis to the severity of the disease [66]. While the immunology of pulmonary fibrosis is still evolving, it is believed to be the end result of chronic inflammatory processes that result in substantial tissue injury. Indeed, the recruitment of macrophages and granulocytes (neutrophils and eosinophils) in the peribronchovascular region can cause substantial lung tissue damage due to the release of reactive oxygen species and toxic granule proteins. In the present study, irrespective of the underlying allergic sensitization, challenge with live but not dead conidia led to increased collagen deposition, which correlated with increased granulocyte recruitment in response to live conidia challenge. While the role of neutrophils in collagen deposition is possibly limited to causing tissue injury [67], eosinophils, macrophages, and Th-2 lymphocytes are likely to play an important role in profibrotic repair, by regulating the recruitment, proliferation, or activation of fibroblasts [68]. Thus, it is possible that waves of leukocytes, with varying functions are recruited in the lung to orchestrate various phases of wound and profibrotic repair after live *A. fumigatus* conidia challenge. Since *ccl17* mRNA was upregulated at day 7 after fungal challenge with live *A. fumigatus* conidia in non-sensitized mice, and neutralization of CCL17 leads to a reduction of bleomycin-induced pulmonary fibrosis [69], it is tempting to hypothesize that upregulation of *ccl17* at day 7 after live *A. fumigatus* challenge marks the shift in host-immune response from a tissue damaging, acute inflammatory response to a profibrotic repair response, and a time-dependent neutralization of *ccl17* may result in reduced pulmonary fibrosis. 

In accordance with the previous study [26], we observed a selective recruitment of neutrophils with inhalation of live *A. fumigatus* conidia but not the dead conidia in the nonsensitized murine host. Previous studies have established the importance of *β*-glucan/dectin-1 and MyD88-dependent signaling pathways in neutrophil recruitment, in response to live *A. fumigatus* challenge in non-sensitized mice [26, 27]. In the present study, the recruitment of neutrophils in response to live and dead *A. fumigatus* conidia in sensitized mice suggests that although neutrophil recruitment requires pathogen associated molecular patterns, it is not exclusive to conidial swelling or live *A. fumigatus *conidia. Increased antigen load or bystander immune activation, in mice sensitized to fungal extracts and challenged with live or dead *A. fumigatus* may result in neutrophil recruitment. Additionally, recruited neutrophils may contribute to increased goblet cell metaplasia seen in these mice (described earlier), via secretion of neutrophil elastase [70–72]. 

In the present study, we identified that neutrophils and eosinophils are recruited with inhalation of live *A. fumigatus* conidia, even in the absence of systemic sensitization (elevated serum IgE) to fungal extracts (non-sensitized host), and without any adjuvant. This is in contrast to a clinically irrelevant, proteinaceous ovalbumin, which is used in many asthma-related animal models and leads to tolerance in the absence of systemic sensitization [73–75]. While several studies have reported the recruitment of granulocytes in response to live *A. fumigatus* conidia [26, 42, 46, 76, 77] and have implicated the role of *β*-glucan, chitin, and secreted proteases, the present study is the first to demonstrate this phenomenon using a natural route of human exposure to dry, aerosolized *A. fumigatus* conidia. Additionally, both eosinophilia [78, 79] and neutrophilia [80–85] have been associated with severe asthma. A subset of asthmatics having eosinophilia and neutrophilia identifies patients with the most severe asthma [86], suggesting the importance of identifying environmental triggers that lead to such inflammation. 

## 5. Conclusion

The differences observed with inhalation of live and dead* A. fumigatus* conidia in non-sensitized mice are pointing towards the emerging conclusion from several different studies that conidial swelling, the first step of germination, is essential in display of immunogenic surface moieties, such as chitin and/or *β*-glucan for the recruitment of eosinophils and neutrophils [26, 28, 29, 36]. Since we administered dry, resting conidia, the swelling of live *A. fumigatus *conidia most likely occurred inside the murine lungs and triggered the recruitment of granulocytes. Dead *A. fumigatus* conidia, due to their incapability to swell and get rid of the immunologically inert surface rodlet layer, probably provide insufficient antigens and/or PAMPs to trigger the immune response. However, sensitized mice, which are predisposed to launch a Th-2-mediated immune response, are hypersensitive to inhaled dead *A. fumigatus *conidia thereby recruiting neutrophils and inducing goblet cell metaplasia, both of which may lead to asthma exacerbation in humans [83, 87–90]. 

A limitation of the present work relates to the fact that fungal dose, exposure frequency, and mouse strain-dependent differences may lead to variations in the extent of allergic and/or hypersensitivity pneumonitis response. Nonetheless, *A. fumigatus* exposure in the environment is ubiquitous, and repeated exposures to high and low doses, at varying frequencies, may occur in different settings, such as in mold infested homes, agricultural settings, or as isolated outdoor exposures. Animal models that mimic natural human exposure to aerosolized fungal spores are critical in investigating the host pathogen interaction, which govern *A. fumigatus*-induced pulmonary disease. Lack of evidence-based risk characterization is the prime reason that despite significant advances in analytical techniques that allow the measurement of cultivable and noncultivable fungi and their components [91], there is no consensus on methodology for monitoring spore concentrations in the environment. For the same reason, results across studies cannot be compared and guidelines or personal exposure limits cannot be established, for assessing fungal exposure. Indeed, studies, such as the one presented here, are likely to aid in establishing evidence-based standards for mold-related exposures and in making informed therapeutic decisions for mold-associated diseases.
